# Comparing Diet and Exercise Monitoring Using Smartphone App and Paper Diary: A Two-Phase Intervention Study

**DOI:** 10.2196/mhealth.7702

**Published:** 2018-01-15

**Authors:** Florence Jimoh, Elizabeth K Lund, Linda J Harvey, Catherine Frost, W James Lay, Mark A Roe, Rachel Berry, Paul M Finglas

**Affiliations:** ^1^ Food Angels UK Ltd Newmarket United Kingdom; ^2^ Independent Consultant Norwich United Kingdom; ^3^ Quadram Institute Bioscience Norwich United Kingdom

**Keywords:** adolescent, smartphone app, diet, exercise, food intake, mobile applications

## Abstract

**Background:**

There is increasing recognition that personalized approaches may be more effective in helping people establish healthier eating patterns and exercise more, and that this approach may be particularly effective in adolescents.

**Objective:**

The objective of this study was to investigate the use of a smartphone app (FoodWiz2) in supporting healthy lifestyle choices in adolescence.

**Methods:**

Participants (N=34: 11 male, 23 female) aged 16-19 years in full- or part-time education were recruited from sixth form colleges, schools, and other further education establishments in Norfolk and Suffolk, United Kingdom, between February and May 2015. Participants recorded food intake and exercise using a paper diary for 4-5 weeks and then used the app for the same duration. Initial nutrition education and general support were provided during the paper diary use, but the app included personalized messages sent in response to app activity. At the end of each study phase, participants completed an online questionnaire to describe their experience of using the paper diary and app.

**Results:**

Record completion declined throughout the study, possibly affected by examination pressure. Food intake data showed increased fruit consumption and significantly reduced consumption of chocolate snacks (*P*=.01) and fizzy drinks (*P*=.002) among participants using the app. Questionnaire responses indicated that the app was generally preferred to the paper diary, in particular, the app was seen as less boring to use (*P*=.03) and more acceptable in social settings (*P*<.001).

**Conclusions:**

This app-based approach has shown the potential for a more effective approach to improving adolescent diet and exercise levels.

## Introduction

A wide range of modern technologies designed to support health and well-being of individuals and specific populations are becoming available and increasingly affordable. This study was designed to investigate the use of a smartphone app (*FoodWiz2*) in supporting healthy lifestyle choices in adolescence. Adolescence is characterized by a period of rapid growth and development; the pubertal increase in height and weight coincides with changes in body composition, such as increased muscle and bone mass and fat deposition in girls [1]. Indeed, energy and nutrient requirements are greater in adolescence than at any other time [2]. The poor quality of many adolescent diets is recognized to be an important issue in relation to a range of short- and long-term health outcomes [3-5]. Recent data from large, cross-sectional surveys indicate that adolescent diets do not meet dietary guidelines in the United Kingdom (Lower Reference Nutrient Intakes) or in Europe (Food-Based Dietary Guidelines, such as the Food Guide Pyramid) [6-8]. In a representative sample of the UK population, adolescents (11-18 years) reported consuming less than the recommended or reference amount of fruits, vegetables, and fiber, whereas saturated fat and sugar were more than the dietary reference value. Indeed, nonmilk extrinsic sugars accounted for 15.3% of total energy of this group compared with a reference of 11% [9]. In Europe, adolescent diets were similarly low in fruits and vegetables, as well as dairy, but high in meat and high-fat products and sweets [6].

Despite the implementation of public health campaigns, adherence to dietary advice is poor across many European countries [10,11]. In particular, it has been reported that adolescent diets may already have an impact on several health parameters [3,12-15], and yet, it is still far from clear which are the most effective approaches to change in lifestyle [16,17]. An important aspect of healthy living is the maintenance of a healthy weight that should be achieved by balancing energy intake with energy expenditure, and results of a recent systematic review indicated that diet, physical activity, and behavioral change interventions are effective in reducing body mass index (BMI) in overweight and obese adolescents [18]. Physical activity is considered an important component of a healthy lifestyle and should be encouraged as much as adherence to a good diet [19,20]. There is also a significant body of evidence to suggest that the effects of being overweight can be ameliorated by exercising regularly [21,22]. Furthermore, diet can improve exercise performance [23]. In general, the children of more highly educated parents exercise more and spend less time in a passive activity, such as computer use or watching television or DVDs [24]. Encouraging exercise as a part of healthy living needs to be particularly targeted at lower socioeconomic groups, as these have been identified as being mainly at risk and hard to reach [25], and the use of modern technologies may well go some way toward achieving this aim [26,27].

Dietary intake can be measured by a range of approaches, such as 24-hour recall, food frequency questionnaires (either self-administered or by interview asking about relatively long-term eating habits based on food groups), food diaries, or duplicate diet measurement. The first 3 approaches rely on using food composition databases, which are continuously being improved for content and accuracy. Of the 3 approaches, arguably the most accurate for measuring current food intake is the food diary method, preferably recording weighed food intake for as many days as possible. The main drawback with this approach is that it is labor-intensive and most people will either modify their diet to save writing down small snacks, record inaccurately, or just give up on recording [28]. It is well documented that completing food diaries, in combination with dietary advice, is associated with weight loss [29], and so, this approach has been widely utilized in helping adults lose weight and improve long-term eating habits. The benefit of this approach over following more extreme diets is that individual nutrient intakes can be monitored, and users can be informed about how well their diet matches with the national nutritional recommendations.

There is an increasing recognition that a personalized approach to nutritional modification may be more effective in helping people establish healthy eating patterns and, combined with encouragement to exercise more, lead to the establishment of better lifestyle habits [30,31]. Several recent studies have investigated the effectiveness of Web-based and smartphone apps in improving adherence to dietary advice in adults [32-38]. For example, 8 out of 42 people completed the app intake diary every day (defined as recording more than 500 kcal per day) in a group of overweight and obese adults using an app called *MyMealMate* over a period of 6 months [33]. However, in 2 parallel groups using either Web-based or paper-based approaches, only 1 or 2 people achieved this. The greater adherence to diary completion in the app group was associated with a reported benefit in terms of ease of use and a greater weight loss. Furthermore, modern technologies are being developed to help teenagers with specific health problems, such as type 1 diabetes [39]. Thus, the use of these technologies in adolescents to support healthy lifestyle choices is considered a potentially useful next step, which has been explored in several new studies [40-42]. *FoodWiz2* has been designed as a part of a European funded study to integrate different technologies to deliver personalized dietary advice to support health and well-being. Unlike some other currently available apps, it includes information on macronutrient content of foods taken from the UK food composition tables [43], allowing nutrient content of fresh and unprocessed foods to be available to the user, while other apps base their analysis on industry databases that may include data from a range of sources, including data from other countries.

Despite the proliferation of health-related apps and their apparent potential in dietary interventions, there is currently limited evidence on the experience of using these self-monitoring tools, and how participants perceive the comparison between novel and traditional methods of dietary assessment. Previous research into smartphone apps, personal digital assistants (PDAs), or short messaging service (SMS) interventions have attempted to assess participant experience, usually through questionnaires, and report on domains such as user satisfaction, patterns of usage, engagement, reasons for like or dislike, helpfulness, and influence on self-efficacy [33,35,44-50]. Studies in adolescent groups have examined the barriers and facilitators of using smartphone apps to record diet [42], using mobile technology (photos, emails, and texts) to record diet [41], and using diet recording apps for weight control [40]. These studies have also explored the effect of using different training methods (face-to-face vs telephone) [41] and the use of technology alone versus technology and counseling [40].

The aim of this study was to assess the ease of use, acceptability, and perceived effectiveness of a smartphone app for the measurement of food intake and exercise in adolescents compared with more traditional paper-based approaches.

## Methods

### Study Design and Ethics

All participants were initially asked to record food intake and exercise using a paper-based diary and then to use a smartphone app to record food and exercise as frequently and as accurately as they found feasible for 4-5 weeks. A 2-phase study design was chosen over a parallel intervention study to avoid introducing potential confounding effects, such as social factors, familiarity with the technology, and individual academic achievement. The study design also avoided potential carry-over effects and disproportionate dropout from the app-to-paper group that may have been associated with a conventional randomized crossover design. Personal and professional contacts were used to involve schools and colleges in the design of the study and to ensure that the protocol was appropriate in an educational environment with minimal disruption to pupils. The study was not blinded or randomized, but each participant was allocated a code for data analysis purposes. The study was scientifically reviewed by the Human Research Governance Committee at the Institute of Food Research, Norwich. The study was conducted according to the guidelines laid down in the Declaration of Helsinki, and all procedures involving human subjects were approved by the Oxford C Ethics Committee managed by the UK Health Research Authority (144/SC/1268).

### Participants and Recruitment

Participants aged between 16 and 19 years, still in full- or part-time education, were recruited from sixth form colleges, schools, and other further education establishments in Norfolk and Suffolk, UK, between February and May 2015. The study was advertised through email and phone calls to education centers, followed by an initial presentation explaining the study first to the head and other interested staff and then to the students. The eligibility criteria were absence of chronic illness or disease (such as serious asthma or diabetes), not pregnant, able to give informed consent, have parental support, able or willing to use a smartphone, able to complete paperwork even with assistance, no eating disorders, no involvement in other research projects or weight management program, and not being related to any member of the study team. An inclusion criterion of BMI not below the second centile line on the BMI chart [51] was chosen as a safety measure to ensure that participants were not likely to fall below the defined healthy BMI range during the study. Signed letters of approval to run the study on each premises were obtained from the relevant school or college head (or other designated responsible person) at all participating schools (*Gatekeeper* approval). Written informed consent was obtained from all participants, and parental consent was also obtained for participants aged less than 18 years at recruitment.

**Figure 1:**
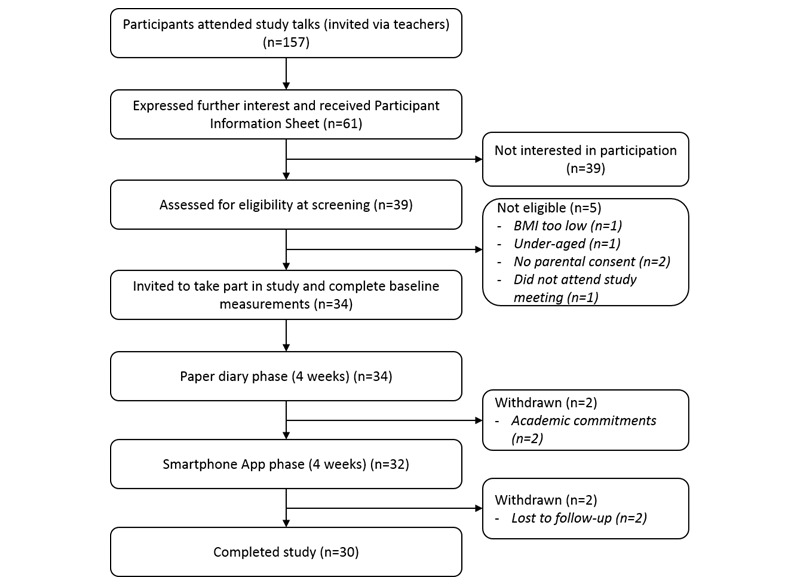
Diagram showing the flow of participants through the study.

Eighteen schools in Norfolk and Suffolk were contacted via email, phone calls, or visit by the researcher. Five schools gave consent for the study talk to be delivered to their students. Recruitment for the study took place between February and May 2015. The flow of participants through the study is described in Figure 1. A total of 157 pupils attended study talks and 38 of these attended the screening session, where participants were assessed for eligibility to take part in the study. Of those screened, 4 were excluded for not meeting the inclusion criteria. Thirty-four adolescents consented and began the paper-diary phase. Two withdrew (the first person after the first week and the second person in the second week of the study) due to academic commitments. Thirty-two participants completed the first phase of the study and were given a smartphone preloaded with the *FoodWiz2* app. Two participants were lost to follow-up during this phase, with 30 completing the study. All 34 participants were, however, included in the analysis of the use of the paper diary, and the 32 that started using the app were included in its analysis.

Following consent, participants were screened based on the eligibility criteria. Weight was measured to the nearest 0.1 kg using a portable electronic scale (Salter Ultimate Accuracy, Tonbridge, Kent, UK); height was measured to the nearest millimeter using a portable stadiometer, and waist circumference was measured to the nearest millimeter using a body waist fitness caliper. Participants then completed a background information form, which provided more information about their diet, previous recording of diet or exercise, use of apps for similar purposes, and educational attainment. A score was calculated for the General Certificate of Secondary Education (GCSE) educational attainment based on a scale of 1-8 for grades G-A (including the higher level A* grade), and a cutoff of 25 (representing grade C in 5 subjects) was used to assess whether there was any difference in the interest in, or ability to, monitor diet among low and high academic achievers. The cutoff of 25 is used in the United Kingdom for assessing suitability at 16 years of age to progress to higher academic studies. Following screening, all participants received nutrition education sessions, covering the basics of a healthy diet, the importance of exercise, and different ways of achieving and maintaining a healthy nutrient intake and net energy intake based on government guidance.

### Recording Diet and Exercise

Participants were initially provided with paper record sheets to record the day, date, time, type, and amount of all foods and drinks consumed; they could also record any recipe in the food record sheets. These food diaries were adapted from those previously used at The Institute of Food Research [52,53]. Participants also recorded the day, date, time type, duration, and intensity of any activity undertaken in the activity sheet. The paper diaries were collected and reviewed with participants every 2 weeks during a face-to-face meeting (10-30 min) between each participant and the researcher. If participants did not attend 2 consecutive meetings with the researchers and were not contactable, they were considered as no longer wishing to take part in the study (lost to follow-up).

After a short interval in recording (1-2 weeks), coincident with a school or college holiday, a smartphone app developed by Food Angels UK Ltd, Newmarket, Suffolk, UK, was given to participants to use for the following 4 weeks (screenshots are available in Multimedia Appendix 1). *FoodWiz2* was developed from an earlier version (*FoodWiz*), which was designed to assist people suffering from allergies. Users of the first *FoodWiz* app can scan the barcode of a product to discover if it contains any allergens of interest (set up via their own personal profile). The more recent *FoodWiz2* app is like other food and exercise recording apps on the market and uses UK measures and databases rather than being US-based. The food composition data used in the app is taken from a commercially available source (BrandBank, Norwich, UK) combined with data for generic foods from the UK McCance and Widdowson’s The Composition of Foods Integrated Dataset [43]. The app allows users to record dietary intake by searching and selecting from a list of foodstuffs and popular meal choices. The user can search by typing in the name of the food or by scanning in barcodes from products. The app also allows users to record intake of home-prepared recipes. A small set of scales, the size of a smartphone, was also provided to allow direct measurement of portion size weights using Bluetooth technology to link the scales to the app [54]. Physical activity could also be recorded using the App, allowing the user to receive instant feedback on their overall daily calorie balance or allowance based on set targets. *FoodWiz2* could also record and track weight and mood of participants. The researcher was able to see the information recorded by each participant using the app via a Web-based portal. Therefore, the researcher could monitor use and send each participant personalized feedback text messages for motivation and help. All participants were made aware that this would happen before the start of the study, both in person and in written form (Participant Information Sheet). Example feedback messages were approved during the ethical review process.

### Ease of Use, Acceptability, and Perceived Effectiveness of the Recording Methods

At the end of each phase of the study, participants were asked to complete an online questionnaire (Multimedia Appendices 2 and 3). The questions were about the pattern of use, what it was like to use the tools, and the perceived impact on dietary and physical activity behavior. Examples of questions asked were, “To what extent do you agree with the following statements” e.g. on the usability (‘was it time-consuming, disrupting, enjoyable, boring, convenient’), acceptability (‘comfortable to use in social setting, would use in future’) and perceived impact of each method of dietary assessment and exercise recording (‘changed portion sizes of meals, ate more fruits and vegetables, increased awareness of physical activity, increased motivation to change physical activity’). An additional questionnaire section was completed at the end of the app phase (Multimedia Appendix 3) to assess the specific features of the app (eg, search function, feedback messages), and a final section compared the 2 recording methods. Questions were scored on the Likert scale, but participants were also given the option of explaining some of their responses in more detail using a free-text approach. Free-text answers were independently reviewed by 2 researchers (FJ and PB), each identifying common topics before agreeing to the most important issues highlighted by participants. The statements included were agreed by both researchers. However, no computer-based systematic analysis of the free-text answers was undertaken due to the limited amount of data available.

### Statistical Analysis

For the analysis of reported food intake using paper diaries or the app, descriptive measures were calculated to describe the sample by use of percentages, means, and standard deviations. Fruits and vegetable servings per day were calculated by dividing the total fruit and vegetable servings consumed by the total number of days that eating occasions were recorded using either the paper diary or App as described by Aflague et al [55]. The number of days a participant used the paper diary or App was set at the total number of days participants recorded food intake of ≥500 kcal.

Two analyses were conducted on questionnaire data because of missing responses: answers to individual questions were described as percentages of all data provided with a subsequent comparison of responses for just those who included their unique identity code, allowing paired analysis of the responses given. The significance of differences in the values of responses to the questionnaire after each phase of the study was evaluated using the sign test for nonparametric paired ordinal data. This test is more conservative than the more frequently used Wilcoxon signed rank test but more appropriate for this dataset where it is difficult to prove the difference between pairs is ordinally scaled [56].

Microsoft Excel 2010 for Windows was used to enter the data, and statistical analyses were performed using SPSS, Version 22 (SPSS Inc., Chicago, IL, USA). All tests were 2-tailed and *P*<0.05 was taken as indicating statistical significance.

## Results

### Participants and Recruitment

Participants (23 female, 11 male) aged 16 to 19 years were recruited in this study (Table 1). The mean participant BMI was 24 kg/m^2^(SD 4) with 15% (5/34) classified as either clinically obese (BMI above the 98th centile based on their sex and age) or severely obese (BMI above 99.6th centile based on their sex and age). All participants remained weight-stable throughout the study with a median weight change of −0.1 kg (interquartile range [IQR]=−0.5 to −0.3, n=33) after the paper diary phase and a median change of 0.375 kg (IQR=−0.275 to −0.925, n=28) after the app phase compared with weight at the start of each phase (overall median study weight change=−0.05 kg; IQR=−1.87 to −0.488; n=30; *P*=.20). Of the 34 participants, 10 females and 1 male had previously recorded their diet or exercise. Six participants (4 female, 2 male) had previously used an app for recording food intake. Average GCSE point score was 40.6 and ranged from 0 to 85, with 58.8% (20/34) of participants scoring 25 and above. All except 3 participants who scored above 25 were attending sixth form schools. Participants who scored zero (n=6) were either registered to take their GCSEs (n=5) or had emigrated from outside of Europe (n=1), where the system of education was different.

### Recording Diet and Exercise

The paper-diary phase was completed by 32 out of the 34 participants who started the intervention, whereas the subsequent App phase was completed by 30 out of the remaining 32 participants. Only 12% (4/34) of participants recorded on all the possible days (28 days in both phases). The mean number of study days completed (>500 kcal) for each 28-day phase is shown in Table 2. Use of the diet recording tool was highest in the paper diary group with a mean of 24 days (SD 6) completed compared with 17 days (SD 9) for the app (*P*=.002). There was no significant difference in completion rate between male and female participants, and no effect of educational attainment on completion of food records was identified at either phase.

The mean percentage completion for the paper diary was 86 (SD 10), whereas that for the app was 61 (SD 7), although it should be noted that most students were taking examinations during the App-based phase. Completion rate decreased gradually throughout the study period both during the paper diary phase and the app phase (Table 2), although use showed a trend toward an increase near the end of the app phase.

**Figure 2:**
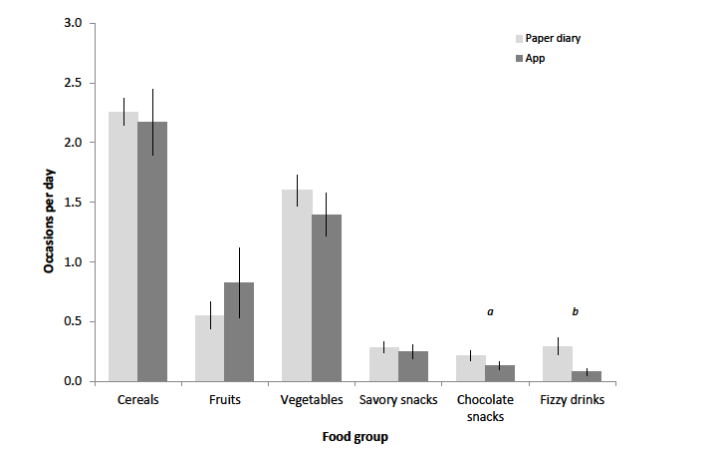
Analysis of records per food group for the paper diary and app. Data are expressed as an average per day on which a total >500 kcal was recorded. Errors are expressed as standard error of the mean (SEM, n= 32). There is a significant difference between the use of paper diaries and the app in the recording of chocolate snacks and fizzy drinks (a: *P*=.012; b: *P*=.002).

Reported food intakes were analyzed by food groups, only for those days where >500 kcal were recorded to allow for comparison between results from the paper diary and the app. In general, recorded food intake was similar using either method; however, the reported consumption of chocolate snacks (*P*=.01) and fizzy drinks (*P*=.002) was significantly lower during the app phase than when paper diaries were used (Figure 2). It was noted by researchers that the quality of the data retrievable from the app was considerably better in terms of specific foods eaten and sources. For example, although a paper diary may just say “Chinese take away,” the app would prompt for a more specific description, for example, “Noodles and sweet and sour chicken.”

All participants recorded a range of exercises in the paper diaries, most frequently walking and cycling but also team sports, gym, dance, and housework. Similarly, all those still in the study used the app for this purpose on at least 1 occasion with 2 participants providing data for at least 26 days, in comparison with 8 participants using the paper diary. Interestingly, there was a significant correlation between the numbers of days completed in the paper diary and the app (*P*<.001), although significantly more days were completed in the paper diary phase with a median of 16 days as compared with 6 days (*P*<.001).

**Table 1:** Characteristics of participants. SD: standard deviation; BMI: body mass index; GCSE: General Certificate of Secondary Education.

Characteristics		Male (n=11)	Female (n=23)
Age (years), mean (SD)		16.8 (0.8)	17.1 (0.85)
Weight (kg), mean (SD)		75.6 (12.76)	66.6 (12.59)
Height (m), mean (SD)		1.8 (0.08)	1.7 (0.06)
Waist circumference (cm), mean (SD)		85.0 (12.88)	79.1 (10.66)
BMI (kg/m^2^), mean (SD)		24.6 (4.21)	24.2 (4.64)
**BMI classification^a^, n**			
	Normal (below the 91st centile)	7	17
	Overweight (above 91st centile)	1	4
	Very overweight or clinically obese (above 98th centile)	2	0
	Severely obese (above 99.6th centile)	1	2
Vegetarian or vegan, n		0	2^b^
Special diet, n		0	1^c^
Allergies, n		0	1^d^
Supplements, n		3^e^	0
Previously recorded diet or exercise, n		1	10
Previous use of diet or exercise app, n		2	4
**Educational attainment classification^f^, n**			
	Below 25 GCSE points	5	9
	25 and above GCSE points	6	14

^a^BMI thresholds vary by sex and one-year increments in age. The age range covered is 2-20 years (Boys UK and Girls UK, Body mass index, 2-20 years [51]).

^b^A participant became vegetarian after 2 weeks in the study (decision independent of the study).

^c^Mild intolerance to wheat and dairy.

^d^Penicillin allergy.

^e^Multivitamins and glucosamine phosphate (n=1); protein occasionally and vitamin tablets in winter (n=1); vitamin D, Branch chain amino acids, whey protein and creatine monohydrate (n=1).

^f^A score was calculated for GCSE based on a scale of 1-8 for grades G-A (including the higher level A* grade). The cutoff of 25 is equal to grade C in 5 subjects.

**Table 2:** Percentage completion of the study diary and App presented by weeks of the study. A completed day was regarded as a day with ≥500 kcal energy recorded [33]. SD: standard deviation.

Week	Percentage completion (SD)	Diet recording tool
1	96 (4)	Paper diet record
2	92 (7)	Paper diet record
3	82 (2)	Paper diet record
4	73 (7)	Paper diet record
5	66 (8)	App
6	61 (4)	App
7	54 (3)	App
8	64 (4)	App

### Ease of Use, Acceptability, and Perceived Effectiveness of the Recording Methods

In most cases, no significant difference in response to Likert scale questions relating to the paper diary and the app were found. The free-text responses gave some additional insight into the issues raised (Multimedia Appendix 4). Although the number and range of responses were limited, they only gave a general indication of opinions. Review of the free-text answers given in the questionnaires suggested an overall preference for the *FoodWiz2* app, particularly in relation to enjoyment, convenience, recommendation to a friend, overall liking, and using again in the future, whereas the paper diary was considered time-consuming and boring. The main reasons given for preferring the app were focused on the topics of usability (eg, “Easy and fun to use”), accessibility (eg, “I can use it anywhere I want”), and the ability to track weight and calorie intake (eg, “Easy to watch calories”). Features of the app which were liked most were the smiley mood scale, bar code scanner, layout, and calorie counting.

The questions relating to patterns of use revealed that the reported level of use of the 2 approaches was similar, although the participants believed they used the paper diary for more days a week than the app (Question 1, Multimedia Appendix 2, *P*=.04), a result consistent with the actual data from the 2 recording methods. Participants reported that the paper diary was bulky and not easy to travel with, which often resulted in them recording what was eaten sometime after consumption. The problems associated with using a paper diary, perhaps only filling it when at home, would appear to have been more than outweighed by potential study fatigue and the issue of exam pressure during the app test phase (Multimedia Appendix 4).

**Figure 3:**
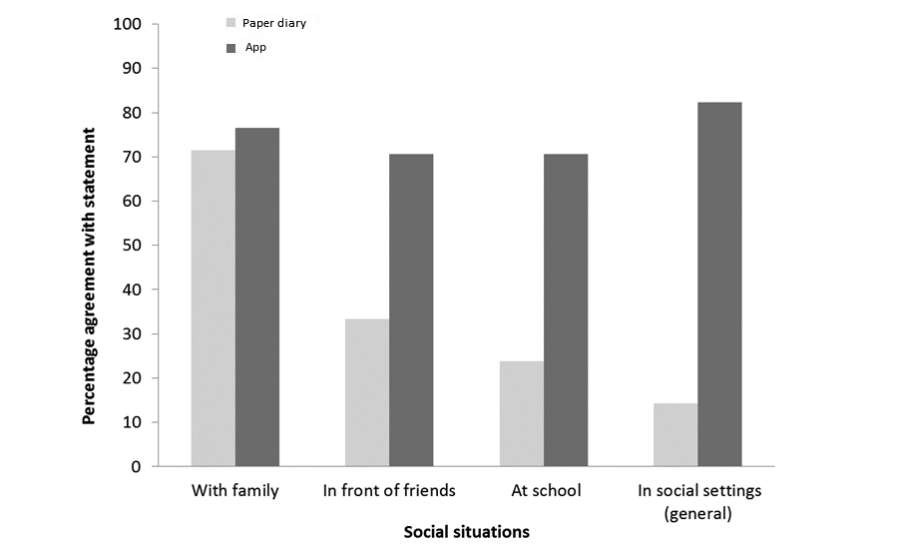
Response to questionnaires in relation to how comfortable participants felt using the paper diary or app in different social scenarios. There is a significant difference between the use of paper diaries and the app in social settings (*P*<.001).

**Figure 4:**
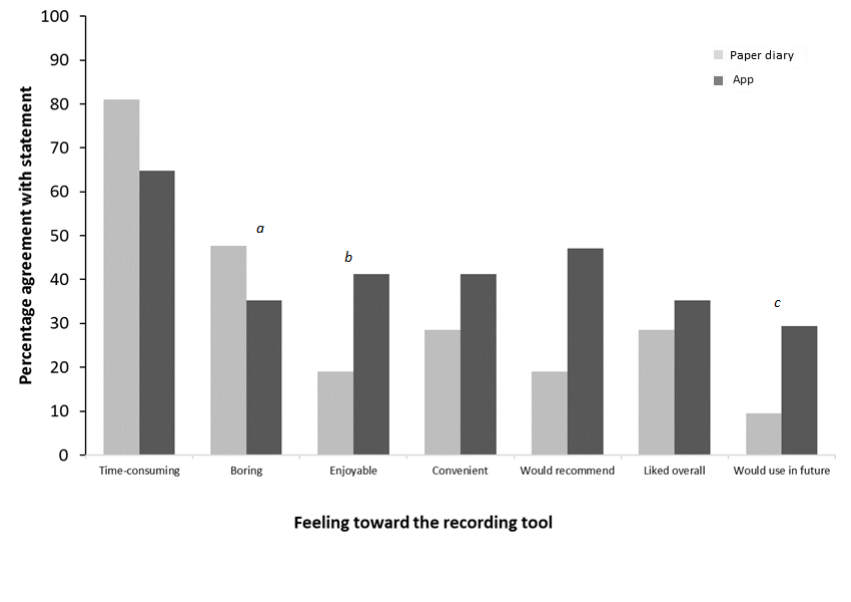
Response to questionnaires in relation to participants’ general feelings towards using either the paper diary or app. For statements a, b, and c, there is a significant difference between the use of the paper diary and the app (a: *P*=.031; b: *P*=.04; c: *P*=.013).

Using both the paper diary and the app were considered time-consuming for different reasons. The paper diary involved having to manually write information and weigh foods, whereas the app had technical issues, for example, it worked slower than anticipated (Multimedia Appendix 4), partially explaining the lack of difference in response to the specific questions as to how time-consuming it was. Furthermore, although free-text answers suggested participants found the app more convenient, no significant difference was identified on the specific question (Section 1b-Q1, Multimedia Appendices 2 and 3). However, participants did report greater social acceptability for using the app (Figure 3). A higher proportion reported that they felt comfortable using their smartphone in social settings compared with using the paper diary (14/17 vs 3/21, *P*<.001). Although, when broken down into more specific social occasions, this effect was no longer significant and very few found using either approach difficult in front of their families. Furthermore, 48% (10/21) reported that they found the paper diary boring, whereas only 33% (5/15) found the app boring (*P*=.03, Figure 4).

Most participants reported that the use of both the paper diary and the app raised awareness of what they had been eating and how active they had been, and they felt that the app was more effective in this respect, which was again consistent with actual recorded data for chocolate snacks and fizzy drinks. However, there were no significant differences in perceived effectiveness between the 2 methods. When indicating the overall preference between the paper diary and the app, 5 out of 12 participants preferred the app, 3 out of 12 preferred the paper diary, and 4 out of 12 had no preference. Participants reported performing more aerobic and strength exercises as well as trying new activities; however, this was not significant, and indeed, the App appeared to significantly reduce confidence to do more activities. This reduction may be explained by the comments about the lack of options in the set list for exercise on the app (Multimedia Appendix 4). Specific questions addressing the quality of the app highlighted the value of being able to search for foods, but potential barriers to the use of the smartphone app were also identified. Feedback from the questionnaire showed that participants needed support with downloading and setting up *FoodWiz2* as it was not available via the usual app download routes (eg, Android Store). Occasionally, participants encountered difficulty finding nongeneric food products, which may have been a limitation of the search function. Suggested improvements to the app included healthier food recommendations, recipe ideas, more physical activity options, and help with portion sizes. The problems encountered with the app were mainly the speed of loading searches and ease of finding specific food items.

## Discussion

### Principal Findings

Adolescence is a key point in life in establishing long-term eating patterns as young people move forward into adult life with growing independence in food choice. Results of this study demonstrated that the app is a potentially feasible method of recording diet and physical activity in adolescents. Previous similar studies using smartphone apps with adults have focused on specific applications such as weight loss or caloric balance [33,49,57], including an intervention to encourage more attentive eating [58], as well as for other clinical issues such as pain management [59,60].

### Strengths and Limitations of the Study

This study had several strengths including the fact that it focused on encouraging overall dietary improvement rather than calorie control in adolescents. This was considered a key issue, as some young people are very sensitive about their weight and may be particularly at risk of developing eating disorders such as anorexia or bulimia [61]. *FoodWiz2* included information from the UK food composition tables, so it was more comprehensive than those using solely commercial brand data sources that do not include products that are not prepacked, for example, fresh fruit, vegetables, and meat. The study also compared the new app technology with the standard paper diary, and participants were able to give feedback on their experience of using either recording method by answering both closed and open questions. The data were considered in the light of recorded food intake data as well as comparing intake data from the app and the paper-based diary. The trial retention in this study was higher (94% [30/32] of participants in the app phase) than that reported by Carter et al [57] testing a different app with women aged 35 years (SD 9). An equal number of participants were lost from the paper diary phase and the *FoodWiz2* app phase and were all linked with academic commitments. Participants were either in full-time or part-time education and so this was not unexpected. The study was subject to several challenges. Recruitment was lower than planned, such that only 57% (34/60) of the original recruitment target was reached. Direct contact with schools was the most successful recruitment strategy, but delays in starting arose as a result of one large potential source of participants withdrawing their support just as recruitment was about to start. This delay meant that the study ran over the normal school examination period in the United Kingdom from May to June. The frequency of use of the interventions was significantly higher in the paper diary compared with the app group (*P*=.002). This was unexpected and is not consistent with what has been previously reported [33,57,62]. This is likely to be due to two factors. First, the participants completed 4 weeks of recording using the paper diary before using the app for the same period and completion consistently dropped over this period, suggesting a fatigue effect. The use of the paper diary declined from the first week, but the use of the app increased slightly in the fourth and final week after exams had finished. The decline in the frequency of use of self-monitoring devices has been previously reported [57,63] in 2 studies comparing adherence to completion of dietary records using smartphone, website, or paper diary in randomized controlled trials. Second, review of the free-text answers highlighted that many participants were under pressure from academic commitments, especially those that had to prepare and appear for their advanced level examinations during the study, which impacted their ability to use the app fully. The comments by the participants that the paper diary was bulky and not easy to travel with resulting in them not recording what was eaten until sometime after consumption is likely to introduce inaccuracy in recording [49,64], although this was not detectable in this study. It is interesting to note that there was no significant difference between males and females or effect of educational attainment on the numbers of days on which food and exercise was recorded, although it should be noted this was a small self-selected subpopulation of all potential participants. The observed similarity in responses between male and female participants contrasts with the results reported in a recent study from the United States with children aged 3-10 years [55].

### Effects of Interventions

Free-text responses in the questionnaires indicated that using either the paper diary or the *FoodWiz2* app raised participants’ awareness of what they were eating and their level of activity. This also seemed to impact participants’ view of their choice of food and the level of activity, particularly when participants used the app. This result is consistent with previous studies, where the recording of diet or activity has led to increased awareness and, in some instances, change in behavior [65-67]. Furthermore, the questionnaire data in this study suggesting that participants believed they had modified their intake of unhealthy foods, albeit not statistically significant, was reflected in the results of the quantitative analysis of food entries in both the paper diary and the app, where significant reductions in chocolate snacks and fizzy drinks were reported using the app. In terms of acceptability and ease of use, participants were more comfortable using the app in different settings, especially in school and in social settings. These results are similar to those of previous studies [57,63]. In these previous studies, a significantly higher proportion of participants reported that the smartphone and website records were convenient to use compared with the paper-based record.

### Conclusions

Our results indicate that, in general, participants preferred the use of a smartphone app to the more traditional paper diary, although some technical issues need to be addressed. In particular, participants found it more comfortable to use the app in social settings. They perceived that the use of the app had more impact on their dietary intake as well as physical activity, compared with the paper diary. Analysis of data from the recorded food intake also showed significantly reduced consumption of chocolate snacks and fizzy drinks among participants when they used the app to record their food intake compared with using the paper diary. The use of mobile technology shows great promise for reducing the burden of self-monitoring lifestyle in this age group, but future apps need to be more sophisticated than the one used for this study. Finally, the relative ease of data extraction for the app compared with coding food diaries and the quality of detail provided mean similar tools show great promise for research purposes.

## Appendix

Multimedia Appendix 1 Screenshots from the FoodWiz2 app. A: the main menu; B: the home screen showing weight, daily targets, and mood; C: adding individual foods and portions.

Multimedia Appendix 2 The study questionnaire used following phase 1 (the paper diary).

Multimedia Appendix 3 The study questionnaire used following phase 2 (the App).

Multimedia Appendix 4 Free-text responses illustrating views expressed by participants responding to the questionnaire with free-text answers.

## Abbreviations

BMI: body mass index
GCSE: General Certificate of Secondary Education
PDAs: personal digital assistants
SD: standard deviation
SMS: short messaging service
