# Supplementary figures and images for: Comparing Diet and Exercise Monitoring Using Smartphone App and Paper Diary: A Two-Phase Intervention Study
Source: JMIR Mhealth Uhealth. 2018 Jan 15;6(1):e17. doi: 10.2196/mhealth.7702 (PMC5789166; doi:10.2196/mhealth.7702)

A

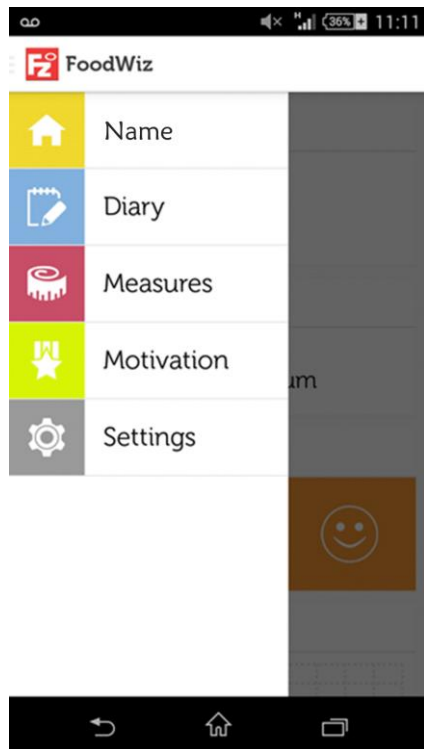

B

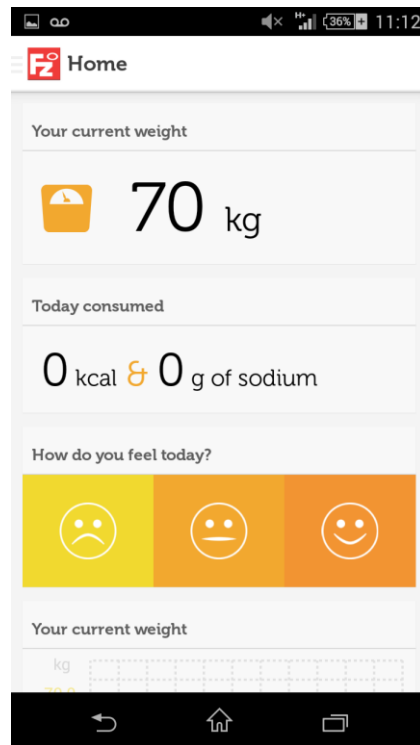

C

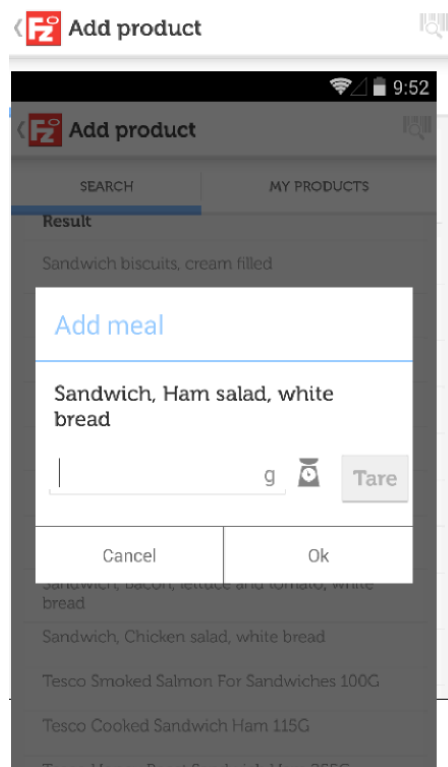

Supplement: Multimedia Appendix 1 [file mhealth_v6i1e17_app1.pdf]
