# Supplementary material for: Comparing Diet and Exercise Monitoring Using Smartphone App and Paper Diary: A Two-Phase Intervention Study
Source: JMIR Mhealth Uhealth. 2018 Jan 15;6(1):e17. doi: 10.2196/mhealth.7702 (PMC5789166; doi:10.2196/mhealth.7702)
Supplement: Multimedia Appendix 2 [file mhealth_v6i1e17_app2.pdf]

Testing the FoodWiz2 App Study

Participant Questionnaire – End of Phase 1 (Paper diary)

As described in the information provided at beginning of the study, we would like you to complete a questionnaire about your experience with using the **paper-dairy** to record your food intake and exercise. The information you provide will help us find out how best to support young people to eat healthy diets and be active. Your responses are confidential and will only be used for survey purposes. It should take about 20-30 minutes of your time. If you have any questions about this questionnaire or experience any technical problems, please contact [researcher name] on [telephone] or [email].

Thank you very much for your time and help.

[ ]	Please enter your volunteer code
[ ]	Please enter the date you completed the questionnaire

Section 1 – Using the paper diary

Section 1A - Patterns of use

1. On average how many days a week did you typically use the paper diary? (Please select one option)

   1   2   3   4   5   6   7

2. On these days how many times did you typically use the paper diary? (Please select one option)

   1-2   3-4   5-6   7-8   8+

3. How many minutes did you typically use the paper diary for each session? (Please select one option)

   1-5mins   6-10mins   10-15mins   15+mins

4. When did you typically use the paper diary to record your **food and drink**? (Please select one option)
   a. During or after each meal or snack? ______
   b. A few times a day? ______
   c. At end of the day? ______
   d. The next day? ______
   e. Once or twice a week? ______
   f. At the end of the week? ______

Comments:____

5. If you left a long time between consuming a food and recording it did you:
    a. Try to remember it and fill it in? _____
    b. Leave it not filled in? _____
6. Did you ever fill your food in in advance? **YES / NO**
   Comments:____

7. How did you typically use the paper diary to record your **physical activity**? (Please select one option)
    a. During or after each activity? _____
    b. A few times a day? _____
    c. At end of the day? _____
    d. The next morning? _____
    e. Once or twice a week? _____
    f. At the end of the week? _____

   Comments:____
8. If you left a long time between doing an activity and recording it would you:
    a. Try to remember it and fill it in? _____
    b. Leave it not filled in? _____
9. Did you ever fill your physical activity in in advance? **YES / NO**
   Comments:____
10. Did you use the paper diary differently at weekends? **YES / NO**
   Comments:____
11. Did the way you use the paper diary change over time? **YES / NO**
   If yes please provide details (select/complete all that apply)
   a) Did how often you used it change: **YES / NO**
      Comments:____
   b) Did the amount of detail you entered change: **YES / NO**
      Comments: ____
   c) Other (please describe): ____
      ____

12. Please indicate how often you think you recorded the following foods/activities

	Never	Not very often	Some of the time	Most of the time	Nearly always
Main meals	◯	◯	◯	◯	◯
Snacks	◯	◯	◯	◯	◯
Drinks	◯	◯	◯	◯	◯
Foods consumed at home	◯	◯	◯	◯	◯
Foods consumed outside the home	◯	◯	◯	◯	◯
Structured activities (e.g. gym session, football)	◯	◯	◯	◯	◯
Regular activities (e.g. walking to school, house work)	◯	◯	◯	◯	◯
Occupational activities (e.g. school)	◯	◯	◯	◯	◯

Please use this space to provide any comments about your answers above:

____

____

____

____

____

Section 1B – What was it like to use the paper diary?

1) To what extent do you agree with the following statements about the paper diary?

	Strongly disagree	Disagree	Not sure	Agree	Strongly agree
Using it was time consuming.	◯	◯	◯	◯	◯
The paper diary did what I wanted it to do.	◯	◯	◯	◯	◯
Using it was disrupting.	◯	◯	◯	◯	◯
Using it was enjoyable.	◯	◯	◯	◯	◯
Using it was boring.	◯	◯	◯	◯	◯
Using it was convenient.	◯	◯	◯	◯	◯
Using it was frustrating.	◯	◯	◯	◯	◯
It is easy to use.	◯	◯	◯	◯	◯
It is easy to learn to use.	◯	◯	◯	◯	◯
I would recommend it to a friend.	◯	◯	◯	◯	◯
I was comfortable using it in social settings.	◯	◯	◯	◯	◯
Over all I liked using the paper diary.	◯	◯	◯	◯	◯
I would use the paper diary again in future.	◯	◯	◯	◯	◯

Please use this space to provide any comments about your answers above:

____

____

____

____

____

2) To what extent do you agree with the following statements about using the paper diary in social situations (if you did not use the paper diary in these situations please select 'N/A').

	N/A	Strongly disagree	Disagree	Not sure	Agree	Strongly agree
I felt comfortable using it in front of my friends	◯	◯	◯	◯	◯	◯
I felt comfortable using it in front of my family	◯	◯	◯	◯	◯	◯
I felt comfortable using it at school	◯	◯	◯	◯	◯	◯

Please use this space to provide any comments about your answers above:

____

____

____

____

Section 2 – Impact on behaviour

Section 2A – Changes in behaviour

1. Did you try to follow the dietary guidelines while using the paper diary?   **YES / NO**

2. Did you try to follow the physical activity guidelines while using the paper diary? **YES/ NO**

3. Please indicate how often you made the following changes while using the paper diary (if you didn't make a change because your intake or level was already good please select 'N/A'):

	N/A	Never	Not very often	Some of the time	Most of the time	Nearly always
I changed the amount of calories I ate	○	○	○	○	○	○
I changed the portion sizes of my meals	○	○	○	○	○	○
I changed the portion size of my snacks	○	○	○	○	○	○
I tried to eat new foods	○	○	○	○	○	○
I swapped some less healthy foods for more healthy ones	○	○	○	○	○	○
I ate more fruits and vegetables	○	○	○	○	○	○
I ate less saturated fat	○	○	○	○	○	○
I ate more fibre	○	○	○	○	○	○
I ate less sugar	○	○	○	○	○	○
I did more aerobic exercises	○	○	○	○	○	○
I did more strength exercises	○	○	○	○	○	○
I tried some new activities	○	○	○	○	○	○
I chose not to eat something because I didn't want to record it	○	○	○	○	○	○
I chose to eat something because I wanted to record it	○	○	○	○	○	○
I chose not to do an activity because I didn't want to record it	○	○	○	○	○	○
I chose to do an activity because I wanted to record it	○	○	○	○	○	○

Please use this space to provide any detail about the changes you made to your diet or physical activity if you wish:

____

____

____

____

____

Section 2B – How helpful was the paper diary?

1) Diet

To what extent do you agree with the following statements about the paper diary?

	Strongly disagree	Disagree	Not sure	Agree	Strongly agree
Using the paper diary increased my awareness of my dietary intake.	◯	◯	◯	◯	◯
Using the paper diary reminded me about my dietary goals.	◯	◯	◯	◯	◯
The paper diary did not help me to learn about my diet.	◯	◯	◯	◯	◯
Using the paper diary increased my motivation to change my dietary intake.	◯	◯	◯	◯	◯
The paper diary did not help me to monitor my diet.	◯	◯	◯	◯	◯
Using the paper diary increased my confidence to change my diet.	◯	◯	◯	◯	◯
Using the paper diary helped me learn about the nutritional content of foods.	◯	◯	◯	◯	◯
Using the paper diary increased my ability to control my dietary intake.	◯	◯	◯	◯	◯
The paper diary did not help me to change my diet.	◯	◯	◯	◯	◯

Please use this space to provide comments on your responses if you wish to:

____

____

____

____

2) Physical Activity

To what extent do you agree with the following statements about the paper diary?

	Strongly disagree	Disagree	Not sure	Agree	Strongly agree
Using the paper diary increased my motivation to change my physical activity.	◯	◯	◯	◯	◯
The paper diary did not help me monitor my physical activity.	◯	◯	◯	◯	◯
Using the paper diary increased my confidence to increase my physical activity.	◯	◯	◯	◯	◯
Using the paper diary helped me learn about the energy used by different activities.	◯	◯	◯	◯	◯
The paper diary did not help me learn about my physical activity.	◯	◯	◯	◯	◯
The paper diary did not help me to change my physical activity.	◯	◯	◯	◯	◯
Using the paper diary increased my awareness of my physical activity.	◯	◯	◯	◯	◯
Using the paper diary reminded me about my physical activity goals.	◯	◯	◯	◯	◯
Using the paper diary increased my ability to control my physical activity.	◯	◯	◯	◯	◯

Please use this space to provide comments on your responses if you wish to:

____

____

____

____

Section 3 – Over all

1) Please tell us what you liked most about using the paper diary:

A. ____

____

B. ____

____

C. ____

____

2) Please tell us if there was anything you didn't like about using the paper diary:

A. ____

____

B. ____

____

C. ____

____

Section 4 – Some more detailed questions you might like to write about

1) **Please write about your experience using the paper diary?** For example what was it like to use it and why? Was it how you expected?

2) **Please write about how you typically used the paper diary?** You might want to think about where, what times, who you were with, what mood you were in. Was there any reason you used it like that?

3) **Can you write about a day when you used it differently or didn't use it?** What was different about that situation? Were there any other situations where you used it differently? Did use change over time?

4) **Tell me about the paper diary itself. What did you think?** How does it compare to other records you've seen? What features were most important to you? What else would you like it to include? What did you like/dislike? Was anything helpful/unhelpful? Easy/difficult?

5) **A) Do you feel the paper diary made a difference to your diet? B) Do you feel the paper diary made a difference to your physical activity?** What changes did you make during the study? How did using the paper diary influence that? Can you give me an example? Have you tried to make changes to your diet and exercise before? Was it different using the paper diary? Can you explain how? What else influenced your diet?

6) **If you were designing a paper diary to help people to eat healthily and exercise, what would you do and why?**
