# Supplementary material for: Comparing Diet and Exercise Monitoring Using Smartphone App and Paper Diary: A Two-Phase Intervention Study
Source: JMIR Mhealth Uhealth. 2018 Jan 15;6(1):e17. doi: 10.2196/mhealth.7702 (PMC5789166; doi:10.2196/mhealth.7702)
Supplement: Multimedia Appendix 4 [file mhealth_v6i1e17_app4.pdf]

Free-text responses illustrating views expressed by participants responding to the questionnaire with free-text answers.

Use characteristics		Paper diary	The App
**Patterns of use**			
	General	“I found it hard to remember to use it”<br>“used when I had time and filled in 2-3 days all in one go”<br>“the paper diary was bulky and not easy to travel”<br>“on weekends I am typically at home which meant I am able to fill it in as I consume or partake in physical activity whereas in weekdays I did not take the diary with me to my 6th form meaning I filled it in either at the end of the day or occasionally at a later date”	“I struggled to keep it as up to date as I should have in the last week due to exams”<br>“I stopped using it when I became too busy”<br>“lots of school work due to exams”<br>“I had to revise for my exams”<br>“[when] I was revising [I] hardly used the app”<br>“really busy or revising”<br>“hardly ever used it, as I have lots of school work due to exams”<br>“I had a period of two weeks for my exams...because I was revising, I hardly used the app”
**Experience using each tool**			
	Time-consuming	“too much detail for example, weight”<br>“I don’t like it because you always need to write everything”<br>“it consumes time, boring and time consuming”	“the app is slow and took ages to load [open]”<br>“the app would be much less time consuming and frustrating if searches were better”<br>“scanner didn’t work”

		"I was unmotivated to use it and ended up just filling most days in before meeting with our group leader for weigh ins"<br>"a lot of details to fill in"<br>"weighing food was a massive [major] issue as well as portion sizes"<br>"it takes time to write what you eat"	"didn't come up with the correct foods"<br>"it was more time consuming than I expected, more so during exams"<br>"it didn't take long to complete"
	Enjoyability		"it was easy to use most of the time and was enjoyable"
	Convenience	"it was harder to record food eaten outside of the home as I did not travel with the paper diary due to it taking up space"<br>"due to largeness [A4 size] I did not want to travel with it or get it out in public"<br>"a minor and obvious problem was the fact that you needed a pen and a suitable surface to fill in data"	"[I liked] being able to add information without any problems"<br>"scanning the barcode [was helpful]"<br>"the option to weigh things via Bluetooth [was good]"
	Social acceptability	"it felt a bit strange and unusual using it in public and around my friend"<br>"I tended to use it at the end of the day"<br>"too embarrassing to use publicly"	"[I] didn't care about filling it in public"<br>"I can use it every time and everywhere"<br>"I can use it anywhere I want"

		"it was always at home and by myself with spare time"	
**Impact on behavior**			
	General	"I found using the paper diary made me more aware of what I was eating"<br>"made me aware of the quantities of food and exercise that I was doing"<br>"lacked any real motivation"<br>"it made you stop and think about unhealthy snacks when you see them on paper"<br>"I have almost completely stopped drinking sugary drinks (choosing water instead) and tend to go for healthier choices when eating in a restaurant"<br>"there was no difference made to my diet as it did not offer any recommendations or way to note how much I was eating in calories, fat or sugar intake"<br>"I was only motivated in fear someone would read it and believe me to be unhealthy"	"the app gave me a lot of motivation to change my diet"<br>"I feel it made a difference to my diet slightly because I saw snacks that were high in calories that I tried to avoid in the future"<br>"make [it made] me more aware of other diets and exercises that I could use and participate in"<br>"I feel more confident to try other foods that I wouldn't normally try due to all the information given alongside using the app"<br>"the app made no difference as I wasn't motivated to use it"
